# Brassinosteroid Ameliorates Zinc Oxide Nanoparticles-Induced Oxidative Stress by Improving Antioxidant Potential and Redox Homeostasis in Tomato Seedling

**DOI:** 10.3389/fpls.2016.00615

**Published:** 2016-05-09

**Authors:** Mengqi Li, Golam J. Ahammed, Caixia Li, Xiao Bao, Jingquan Yu, Chunlei Huang, Hanqin Yin, Jie Zhou

**Affiliations:** ^1^Department of Horticulture, Zijingang Campus, Zhejiang UniversityHangzhou, China; ^2^Zhejiang Provincial Key Laboratory of Horticultural Plant Integrative BiologyHangzhou, China; ^3^Key Laboratory of Horticultural Plants Growth, Development and Quality Improvement, Agricultural Ministry of ChinaHangzhou, China; ^4^School of Environmental Studies, China University of GeosciencesWuhan, China; ^5^Geological Research Center for Agricultural Applications, China Geological SurveyHangzhou, China; ^6^Zhejiang Institute of Geological SurveyHangzhou, China

**Keywords:** brassinosteroids, food safety, hydrogen peroxide, nanotoxicity, oxidative stress, tomato, zinc oxide nanoparticles

## Abstract

In the last few decades use of metal-based nanoparticles (MNPs) has been increased significantly that eventually contaminating agricultural land and limiting crop production worldwide. Moreover, contamination of food chain with MNPs has appeared as a matter of public concern due to risk of potential health hazard. Brassinosteroid has been shown to play a critical role in alleviating heavy metal stress; however, its function in relieving zinc oxide nanoparticles (ZnO NPs)-induced phytotoxicity remains unknown. In this study, we investigated the potential role of 24-epibrassinolide (BR) in mitigating ZnO NPs-induced toxicity in tomato seedlings. Seedling growth, biomass production, and root activity gradually decreased, but Zn accumulation increased with increasing ZnO NPs concentration (10–100 mg/L) in growth media (½ MS). The augmentation of BR (5 nM) in media significantly ameliorated 50 mg/L ZnO NPs-induced growth inhibition. Visualization of hydrogen peroxide (H_2_O_2_), and quantification of H_2_O_2_ and malondialdehyde (MDA) in tomato roots confirmed that ZnO NPs induced an oxidative stress. However, combined treatment with BR and ZnO NPs remarkably reduced concentration of H_2_O_2_ and MDA as compared with ZnO NPs only treatment, indicating that BR supplementation substantially reduced oxidative stress. Furthermore, the activities of key antioxidant enzymes such as superoxide dismutase (SOD), catalase, ascorbate peroxidase and glutathione reductase were increased by combined treatment of BR and ZnO NPs compared with ZnO NPs only treatment. BR also increased reduced glutathione (GSH), but decreased oxidized glutathione (GSSG)] and thus improved cellular redox homeostasis by increasing GSH:GSSG ratio. The changes in relative transcript abundance of corresponding antioxidant genes such as *Cu/Zn SOD, CAT1, GSH1*, and *GR1* were in accordance with the changes in those antioxidants under different treatments. More importantly, combined application of BR and ZnO NPs significantly decreased Zn content in both shoot and root of tomato seedlings as compared with ZnO NPs alone. Taken together, this study, for the first time, showed that BR could not only improve plant tolerance to ZnO NPs but also reduce the excess zinc content in tomato seedlings. Such a finding may have potential implication in safe vegetable production in the MNPs-polluted areas.

## Introduction

Nanoparticles (NPs) are particles that have at least one dimension less than 100 nm; but have a greater surface area compared to bulk products. In the recent years, engineered NPs are extensively being used for manufacturing variety of industrial, commercial, and medical products that are eventually released to the environment. Thus extensive use of NPs has become a matter of public concern due to potential contamination of food chain by metal-based NPs (MNPs). Zinc oxide NPs (ZnO NPs) are one of the MNPs that are commonly investigated with regards to human and ecosystem health as well as nanotoxicological effect on plants (Chen et al., 2015; Van Aken, 2015; Zhang et al., 2015). Given that ZnO NPs are wide band-gap semiconductors that exhibit near UV emission and transparent conductivity, their application as electronic sensors and solar voltaic has become common (Lin and Xing, 2008; Ma et al., 2013). Moreover, ZnO NPs are widely exploited for their photolytic properties and are also extensively used in personal care products for their ultraviolet-blocking ability (Hoffmann et al., 1995; Ali et al., 2008). Earlier studies have already reported the presence of ZnO NPs in sewage treatment plant eﬄuents and in sludge-treated soils used for agriculture (Ma et al., 2013). Another potential formulation of ZnO NPs for agricultural applications could be as a pesticide because of their antimicrobial properties (He et al., 2011; Dimkpa et al., 2013).

Earlier studies on nanotoxicity in plants showed differential effects of MNPs that include positive, negative or no effects on plants (Dietz and Herth, 2011). Nonetheless, most of those studies addressed easily observable parameters, such as germination rate and growth-related features. Plant responses to MNPs not only depend on dose, but also on the species of plants (Chichiriccò and Poma, 2015). For instance, ZnO NPs caused a dose-dependent inhibition in seed germination of cabbage (*Brassica oleracea* var. *capitata* L.), while it showed no negative effects on germination of maize (*Zea mays* L.) seeds (Pokhrel and Dubey, 2013). Moreover, ZnO NPs (1,000 mg/L) reduced root length of corn and cucumber (*Cucumis sativus* L.), but exhibited no effects on their seed germination (Zhang et al., 2015). In comparison with Zn^2+^, toxicity of ZnO NPs on the root elongation of corn could be attributed to the nanoparticulate ZnO, while released Zn ion from ZnO could solely contribute to the inhibition of root elongation in cucumber (Zhang et al., 2015).

On the basis of NP size, shapes, compositions and atomic arrangement, interaction of NPs with cellular structure varies a lot and yet very complex and poorly understood (Van Aken, 2015). Moreover, studies on the mechanism of interaction between NPs such as ZnO NPs and plant cell biomolecules are scanty. A few studies on NPs ecotoxicity suggested several potential mechanisms through which ZnO NPs cause damage to plants (Chen et al., 2015; Zhang et al., 2015). MNPs enter plant tissues, predominantly through pores in the cell wall and endocytosis pathway, and then are translocated through vascular system (Chichiriccò and Poma, 2015). First, the release of Zn^2+^ from ZnO NPs possibly causes phytotoxicity (Lin and Xing, 2008). Second, ZnO NPs due to their small sizes and large surface area may directly interact with biomolecules and disrupt membranes or DNA (Ma et al., 2013). Most importantly, ZnO NPs promote the generation of reactive oxygen species (ROS) such as superoxide radical ($O_{2}^{•–}$) and hydrogen peroxide (H_2_O_2_) in the absence of photochemical energy (Hernandez-Viezcas et al., 2011). It is suggested that excessive generation of ROS can induce membrane lipid peroxidation and cellular damage, which is considered as one of the primary reasons contributing to nanotoxicity in plants (Chichiriccò and Poma, 2015). Plants possess a well equipped antioxidant systems composed of both enzymatic and non-enzymatic antioxidants to scavenge excessive level of ROS that are injurious to cells (Baxter et al., 2014). Exposure of plants to NPs also stimulates antioxidant system in plants, perhaps as an adaptive response to alleviate oxidative stress.

In general, Zn is required for various metabolic processes in human. However, daily intake that exceeds recommended dietary allowance may cause Zn toxicity (Fosmire, 1990). Ingestion of excess Zn causes symptoms like nausea, vomiting, epigastric pain, abdominal cramps, and diarrhea in human. Furthermore, the use of Zn supplements can interfere with the utilization of copper, impair immune function and severely affect lipoprotein profiles (Fosmire, 1990). Vegetables are an integral part of human diet that serves as a major route for intake of essential and/or non-essential metals. An earlier study conducted in a metal-polluted area (Zinc Plant in Huludao City, China) showed high transfer factor values of Cd, Zn, and Cu from soil to vegetable (Zheng et al., 2007). Authors of that study calculated total metal target hazard quotients due to consumption of vegetables, which indicated highest health risks to inhabitants close to Zinc plant. Thus, extensive utilization of ZnO NP and deliberate release to environment may increase possible health risks to population of metal-polluted areas through the food chain transfer. Therefore, elucidation of strategies that improve plant tolerance to MNP as well as reduce metal uptake are urgent to ensure food security and food safety. Till date, studies relating to alleviation of ZnO NPs-induced phytotoxicity are scanty. Recently, Chen et al. (2015) reported that nitric oxide (NO), a ubiquitous signal molecule in plants could ameliorate ZnO NPs-induced phytotoxicity in rice seedlings. A number of earlier studies relating to enhancement strategies of plant tolerance against heavy metal stress (not MNPs) revealed that signal molecule including phytohormones could alleviate heavy metal-induced phytotoxicity in a range of plant species (Ahammed et al., 2014). For instance, (Ramakrishna and Rao, 2012, 2015) reported that 24-epibrassinolide (a steroidal phytohormone) could alleviate Zn^2+^ stress by improving antioxidant potential and redox state in radish (*Raphanus sativus* L.) seedlings. ZnO NPs can also induce Zn^2+^ stress by releasing Zn ions. Thus it is quite likely to anticipate a protective role of brassinosteroids (BRs) against MNPs too.

Brassinosteroids are a class of plant-specific essential steroidal hormones that regulate broad aspects of plant growth, development and responses to various biotic and abiotic stresses (Cui et al., 2011; Ahammed et al., 2014; Zhou et al., 2014; Divi et al., 2016). However, the mechanisms that control BRs-induced stress tolerances are largely unknown (Divi et al., 2016). We previously showed that BRs-induced oxidative stress tolerance involves transient accumulation of hydrogen peroxide (H_2_O_2_) that activates antioxidant system in cucumber and tomato plants (Cui et al., 2011; Nie et al., 2013; Zhou et al., 2014). Although BRs initially induce NADPH-based H_2_O_2_ production, it eventually enhances ROS scavenging by stimulating antioxidative machinery, indicating a dual role of H_2_O_2_ in mediating BR-induced stress tolerance (Ahammed et al., 2014). We also showed that BRs could efficiently ameliorate cadmium (a major toxic heavy metal)-induced oxidative stress and photosynthetic inhibition in tomato plants (Ahammed et al., 2013). However, the effect of BRs on MNPs-induced phytotoxicity still remains elusive. In the current study, we investigated the potential role of 24-epibrassinolide (BR, a bioactive BRs) in mitigating ZnO NPs-induced toxicity in tomato seedlings. This study is expected to provide a better insight into the role of BRs in MNPs-induced oxidative stress that may be useful to ensure food safety in MNPs-polluted areas.

## Materials and Methods

### MNPs Preparation

The ZnO NPs (diameters varying between 20 and 30 nm, a purity > 99%) were purchased from the Aladdin Corporation (Shanghai, China). Culture dispersion of NPs was achieved by adding Phytagel (Sigma–Aldrich, St. Louis, MO, USA) powder and a suitable amount of NPs to ultrapure water, and the dispersions were sufficiently shaken after sonication to break up agglomerates. Each concentration of NPs treatment was prepared separately, without dilution, by weighing particles and dispersing them in solid half strength Murashige and Skoog (½ MS) medium. The addition of NPs ranged from 10 to 100 mg/L. It is noteworthy to mention that the agar culture medium has the advantage of easy dispersion of NPs without precipitation.

### Plant Materials and Treatments

Authentic tomato seeds (*Solanum lycopersicum* L. cv. Hezuo903)were sterilized in a 10% sodium hypochlorite solution for 15 min, rinsed thoroughly with deionized water several times, and subsequently placed in sterilized solid ½ MS medium at a controlled temperature of 28°C in the dark. After 48 h, the seeds were checked for the germination, and seeds that had sprouted were used in the test. The toxicity tests were conducted in a tissue culture bottle (240 mL). Each test unit contained 40 mL of ½ MS culture media with a specific concentration of ZnO NPs. Ten tomato seedlings were placed just above the surface of the medium of the test units. The test units were placed in a sterile room. After an incubation period of 15 days, the plants were separated from the agar media, and seedling growth was measured. The NP concentrations of 0, 10, 20, 50, and 100 mg/L were prepared in four replicate test units per treatment.

In the BR treatment, the 5 nM 24-epibrassinolide was added in the ½ MS medium in the process of the solidification. The concentration of BR was chosen based on preliminary dose trial (data not shown).

### Measurement of Morphological Parameters and Root Activity

Morphological parameters such as fresh weight and length of shoot and root, and root activity were determined following exposure of tomato seedlings to different levels of ZnO NPs for 15 days. In each replicate, 10 plants were selected randomly, and length and fresh weight of each plant were determined. Average values of these 10 plants were considered as one replicate. For determination of root activity, three replicates for each ten treatments such as CK, 10, 20, 50, and 100 mg/L ZnO NPs were selected. Roots were washed thoroughly with distilled water and finally with deionized water and cut into small pieces of 3–4 mm. A 0.5 g portion of these roots sample was placed into tube; 5 mL 0.4% TTC (triphenyl tetrazolium chloride) and 5 mL 0.1 mM phosphatic buffer solution (pH 7.0) were added to the tube and allowed to react for 2 h at 37°C. Afterward, 2 mL of 1 M H_2_SO_4_ was added to the tube to stop the reaction. The root activity was expressed by the amount of TPF (triphenyl formazan) deoxidized by TTC (Islam et al., 2007).

### Determination of H_2_O_2_ Contents and Histochemical Detection of H_2_O_2_

To determine the H_2_O_2_ concentration, 0.3 g of fresh root tissues was homogenized in 3 mL of precooled HClO_4_ (1.0 M) using a pre-chilled mortar and pestle, according to the method of Willekens et al. (1997).

H_2_O_2_ production in root tissues was monitored using 2,7-dichlorofluorescein diacetate (H_2_DCF-DA) as described by Yi et al. (2015). Detached roots were washed with deionized water and incubated 15 min with 25 μM H_2_DCF-DA in 200 mM phosphate buffer (pH 7.4) and then washed five times with the same buffer without the dye. To scavenge H_2_O_2_, the root segments were incubated with 1 mM ascorbate or 100 U/mL catalase for 30 min that served as negative controls. Fluorescence was observed using a Leica DM4000B microscope and images were captured using a Leica DFC425C camera and the Leica application suite V3.8 software (Leica Microsystems, Wetzlar, Hessen, Germany).

### Determination of Lipid Peroxidation

To determine level of lipid peroxidation in roots, concentration of malondialdehyde (MDA) was measured by the 2-thiobarbituricacid (TBA) test. Root samples (0.5 g) were homogenized in 5 mL of 10% (w/v) trichloroaceticacid (TCA). The homogenates were centrifuged at 3,000 *g* for 10 min and 4 mL of 20% TCA containing 0.65% (w/v) TBA was added to 1 mL of the supernatant. The mixtures were heated in a hot water bath at 95°C for 25 min and immediately placed in an ice bath to stop the reaction. After centrifugation at 3,000 *g* for 10 min and the absorbance of the supernatants was recorded at 440, 532, and 600 nm. The MDA equivalents were calculated according to Hodges et al. (1999).

### Determination of Antioxidant Enzyme Activity

Antioxidant enzymes were extracted by grinding the root tissue (0.3 g) with 3 mL ice-cold 50 mM phosphate buffer (pH7.8) containing 0.2 mM EDTA and 2% polyvinylpyrrolidone (w/v). The homogenates were centrifuged at 4°C for 20 min at 12,000 *g*, and the resulting supernatants were used for the determination of enzymatic activity.

Superoxide dismutase (SOD) activity was assayed by inhibiting the photochemical reduction of nitro blue tetrazolium (NBT) according to Stewart and Bewley (1980). The absorbance was monitored in 560 nm. One unit of SOD is the amount of extract that gives 50% inhibition of the reduction rate of NBT. Catalase (CAT) activities were determined following the methods of Cakmak and Marschner (1992). The reaction mixture for CAT consisted of 25 mM phosphate buffer (PH 7.0), 10 mM H_2_O_2_ and enzyme extract. The decomposition of H_2_O_2_ determined at 240 nm for 20 s (*E*_240_ = 39.4 mM^-1^ cm^-1^). Ascorbate peroxidase (APX) was measured in a reaction mixture containing 25 mM phosphate buffer (PH 7.0), 5 mM ascorbic acid (ASA), 20 mM H_2_O_2_ and enzyme extract with the principle of monitoring the rate of ascorbate oxidation at 290 nm for 20 s (*E*_290_ = 2.8 mM^-1^ cm^-1^), according to Nakano and Asada (1981). Glutathione reductase (GR) activity was determined according to Foyer and Halliwell (1976). The reaction mixture for GR contained 25 mM phosphate buffer (PH 7.0), 10 mM glutathione and oxidized glutathione (GSSG), 2.4 mM NADPH and enzyme extract. The detection was based on the rate of decrease in the absorbance of NADPH at 340 nm for 20 s (*E*_340_ = 6.2 mM^-1^ cm^-1^). All of the spectrophotometric analyses were performed using a SHIMADZU UV-2410PC spectrophotometer (Japan).

### Determination of Glutathione

Glutathione contents were determined according to Sgherri and Navari-Izzo (1995). Root tissues (0.3 g) were homogenized in 2 mL of 6% metaphosphoricacid containing 2 mM EDTA. The homogenates were then centrifuged at 4°C for 10 min at 14,000 *g*. The total GSSG contents were determined using the 5, 5′dithio-bis (2-nitrobenzoicacid)-GSSG reductase recycling method. The reaction mixture for total glutathione (GSH+GSSG) consisted of 100mM phosphate buffer (PH 7.5), 6 mM 5, 5′dithio-bis (2-nitrobenzoicacid; DTNB), 0.2 mM NADPH, three units of GR and extract with the principle of monitoring the decomposition of DTNB at 412 nm for 1 min. For detection of GSSG, GSH in the extract was blocked out by adding 2-ethenylpyridine, and then measured following the same procedure of total glutathione. Reduced glutathione (GSH) content was then calculated by deducting GSSG from total glutathione (GSH+GSSG).

### RNA Isolation and RT-PCR

Plant root samples were ground in liquid nitrogen and the total RNA was isolated according to the manufacturer’s protocol using the Trizol reagent (Invitrogen, California, CA, USA). The genomic DNA was removed using the RNeasy Mini Kit (Qiagen, Beijing, China). Total RNA (1 μg) was reverse-transcribed using the ReverTra Ace qPCR RT Kit (Toyobo, Japan) following the manufacturer’s instructions. Gene-specific primers for the quantitative real time PCR (qRT-PCR) were designed based on the mRNA or EST sequences for the corresponding genes as follows: *Cu/Zn-SOD* (F: 5′-GGCCAATCTTTGACCCTTTA-3′, R: 5′-AGTCCAGGAGCAAGTCCAGT-3′), *cAPX* (F: 5′- TCTGAATTGGGATTTGCTGA-3′, R: 5′-CGTCTAACGTAGCTGCCAAA-3′), *GR1* (F: 5′-TTGGTGGAACGTGTGTTCTT-3′, R: 5′-TCTCATTCACTTCCCATCCA-3′), *CAT1* (F: 5′-TGATCGCGAGAAGATACCTG-3′, R: 5′-CTTCCACGTTCATGGACAAC-3′), and *GSH1* (F: 5′-CTGCATTCTGGGTGGGT-3′, R: 5′-CTCGGCTACTTCGTTCA-3′); *Actin* (F: 5′-TGGTCGGAATGGGACAGAAG-3′, R: 5′-CTCAGTCAG-GAGAACAGGGT-3′) was used as an internal control. For the qRT-PCR, the PCR products were amplified in triplicate using the SYBR Green PCR Master Mix (Takara, Tokyo, Japan) in 25 μL qRT-PCR reactions in an iCycler iQ^TM^ 96-well real-time PCR detection system (Bio-Rad, Hercules, CA, USA). The PCR conditions consisted of denaturation at 95°C for 3 min followed by 40 cycles of denaturation at 95°C for 30 s, annealing at 58°C for 30 s and extension at 72°C for 30 s. The software that was provided with the PCR system was used to calculate the threshold cycle values and quantify the mRNA levels according to Livak and Schmittgen (2001).

### Determination of Zn Content

Dry samples (0.10 g) of shoot or root (homogenized and powdered) were digested with a mixture of HClO_4_ and HNO_3_ (v/v = 1/9) at 180°C. The digested colorless liquids were washed three times with distilled water. The liquid was collected and transferred to 50 mL volumetric flasks and diluted to a constant volume. Total Zn concentration was analyzed using an atomic absorption spectrophotometer (AA-6300; Shimadzu Co. Kyoto, Japan) as described by Wu et al. (2005).

### Statistical Analysis

At least four independent replicates were used for each determination, and the mean values of all of the data are presented for each treatment. A statistical analysis of the bioassays was performed with the SPSS 16.0 statistical software package, and a Tukey’s test (*P* < 0.05) was performed to evaluate the treatment effects.

## Results

### Dose Effect of ZnO NPs on Growth and Root Viability of Tomato Seedlings

**FIGURE 1:**
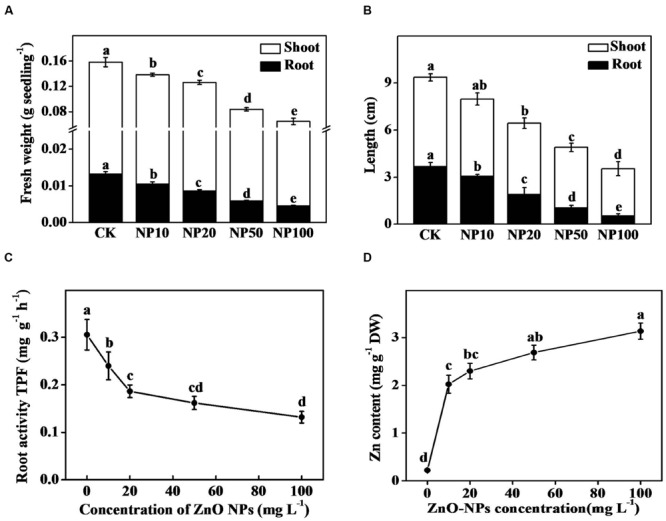
**Effect of different concentration of zinc oxide nanoparticles (ZnO NPs) on growth and biomass accumulation in tomato seedlings. (A)** Fresh weight, **(B)** length of shoot and root, **(C)** root activity, and **(D)** Zn content in seedlings. Tomato seeds after sprouting were placed on ½ MS medium containing graded levels ZnO NPs and cultured for 15 days in tissue culture bottle. Data are the means of four replicates, with standard errors indicated by the vertical bars. Means denoted by the same letter did not significantly differ at a *P* < 0.05, according to Tukey’s test. CK, control; DW, dry weight; NP10, 10 mg/L ZnO NPs; NP20, 20 mg/L ZnO NPs; NP50, 50 mg/L ZnO NPs; NP100, 100 mg/L ZnO NPs; TPF, triphenyl formazan.

To assess toxic effect of ZnO NPs on seedling growth, we first carried out a dose-trial of ZnO NPs by exposing tomato seedlings to different levels of ZnO NPs (10, 20, 50, and 100 mg/L) for 15 days. As shown in **Figures 1A,B**, both fresh biomass and length of shoot and root of tomato seedlings decreased significantly with increasing concentration of ZnO NPs in growth media. In addition, the root activity also reduced gradually with an increase in the concentration of ZnO NP (**Figure 1C**). To understand potential link between exposure concentration of ZnO NPs and subsequent growth inhibition, we quantified Zn content in tomato seedlings. Data showed that Zn content consistently increased with increasing concentration of ZnO NPs in media (**Figure 1D**), indicating that Zn has been absorbed by plant from media in a dose-dependent manner that caused substantial growth inhibition in tomato seedlings.

### BR Improves Seedling Growth and Biomass Accumulation under ZnO NPs Stress

**FIGURE 2:**
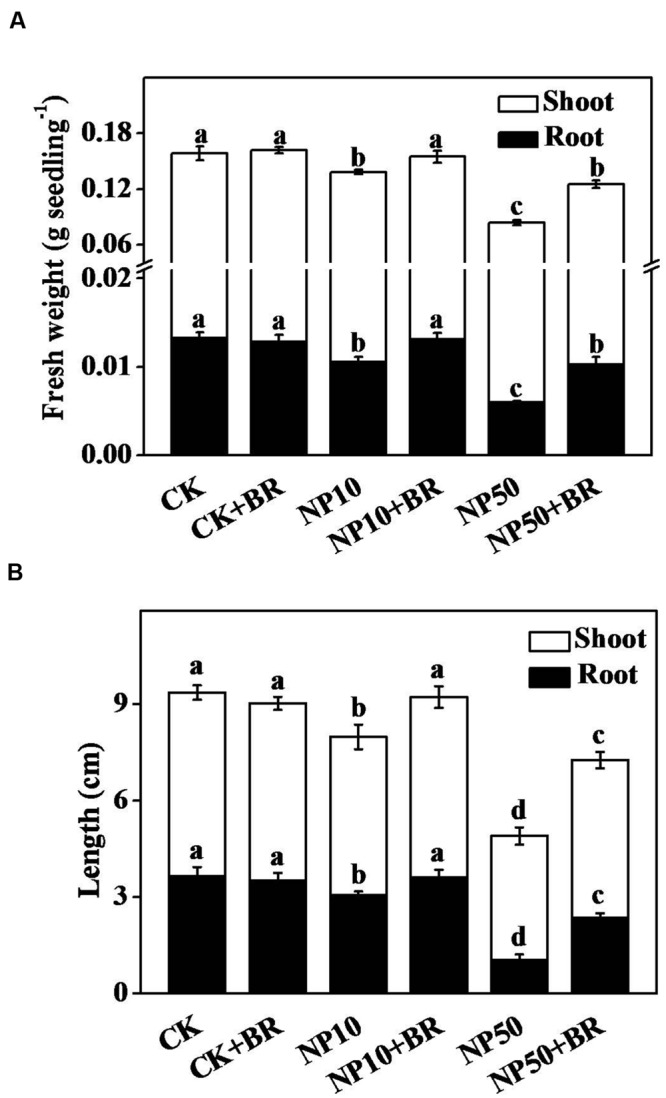
**Effect of brassinosteroid on growth and biomass accumulation under zinc oxide nanoparticles (ZnO NPs) stress. (A)** Fresh weight, and **(B)** Length of shoot and root of tomato seedlings after 15 days culture on the ½ MS culture medium containing 5 nM 24-epibrassinolide (BR) and/or 10 and 50 mg/L ZnO NPs. The data are the means of four replicates, with standard errors indicated by the vertical bars. Means denoted by the same letter did not significantly differ at a *P* < 0.05, according to Tukey’s test. NP10, 10 mg/L ZnO NPs; NP50, 50 mg/L ZnO NPs.

To investigate potential role of BR in tomato ZnO NPs tolerance, we selected two concentrations of ZnO NPs (10 and 50 mg/L) for further study, considering them as low and high dose of ZnO NPs based on our preliminary experiment. As shown in **Figure 2** both low and high concentration of ZnO NPs caused significant reduction in biomass accumulation accounting for 20.7/11.9 and 55.2/46.3% reduction in shoot/root, respectively (**Figure 2A**). Likewise, length of shoot and root decreased by 71.6 and 32.3%, respectively, following exposure of seedlings to 50 mg/L ZnO NPs (**Figure 2B**). In contrast, combined treatment of BR (5 nM) and ZnO NPs remarkably increased fresh weight and length of shoot and root compared with those of ZnO NPs (both 10 and 50 mg/L) only treatments (**Figure 2**). For instance, BR treatment with 50 mg/L ZnO NPs increased fresh weight and length (shoot/root) by 72.4/47.7 and 126.9/26.9%, respectively, compared with those of 50 mg/L ZnO NPs alone. These observations clearly indicate that exogenous BR (5 nM) can alleviate both low and high concentration of ZnO NPs-induced inhibition of growth and biomass accumulation in tomato seedlings.

### BR Minimizes ZnO NPs-Induced H_2_O_2_ Accumulation and Lipid Peroxidation

**FIGURE 3:**
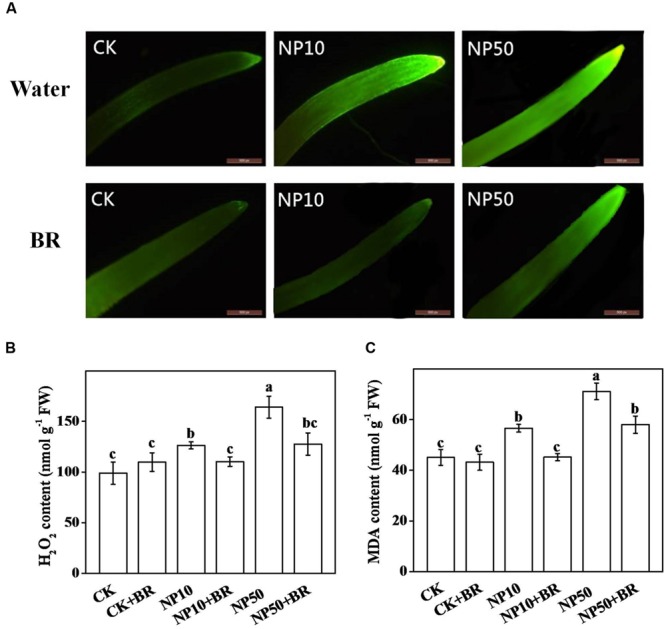
**H_2_O_2_ accumulation and lipid peroxidation in response to brassinosteroid and/or zinc oxide nanoparticles (ZnO NPs) treatments. (A)** *In situ* detection of H_2_O_2_ using H2DCF-DA staining, Bar = 200 μm, **(B)** H_2_O_2_ contents, and **(C)** malondialdehyde (MDA) contents in seedling roots after 15 days culture in the ½ MS medium containing 10 and 50 mg/L ZnO NPs with or without 5 nM 24-epibrassinolide (BR). The data are the means of four replicates, with standard errors indicated by the vertical bars. Means denoted by the same letter did not significantly differ at a *P* < 0.05, according to Tukey’s test. CK, control; FW, fresh weight; NP10, 10 mg/L ZnO NPs; NP50, 50 mg/L ZnO NPs.

To understand potential mechanism of ZnO NPs-induced phytotoxicity, we examined H_2_O_2_ accumulation and lipid peroxidation that are often considered as reliable biomarkers of oxidative state. Hitochemical staining with H_2_DCF-DA showed that exposure of seedlings to both concentration of ZnO NPs increased H_2_O_2_ accumulation in roots as evident by remarkably increased green fluorescence compared with that of control (**Figure 3A**). Biochemical analysis of H_2_O_2_ showed that H_2_O_2_ concentration increased by 27.8 and 66.0% in 10 and 50 mg/L ZnO NPs treatment, respectively, compared with that of control (**Figure 3B**). When BR was added with ZnO NPs in media, accumulation of H_2_O_2_ was reduced significantly compared with that of ZnO NPs only treatments (**Figure 2B**). Interestingly, BR application on non-stressed plants (CK) had no significant effect on H_2_O_2_ accumulation in roots of tomato seedlings (**Figure 2B**). We also quantified the MDA content to evaluate level of lipid peroxidation. In accordance with H_2_O_2_ accumulation, ZnO NPs (low and high) significantly increased MDA content in roots (**Figure 3C**). Compared with ZnO NPs only treatments, MDA content was remarkably decreased when BR was added with ZnO NPs in media, indicating that BR could efficiently minimize lipid peroxidation by lowering ROS accumulation in roots.

### BR Improves Antioxidant Enzyme Activities and Redox Homeostasis under ZnO NPs Stress

**FIGURE 4:**
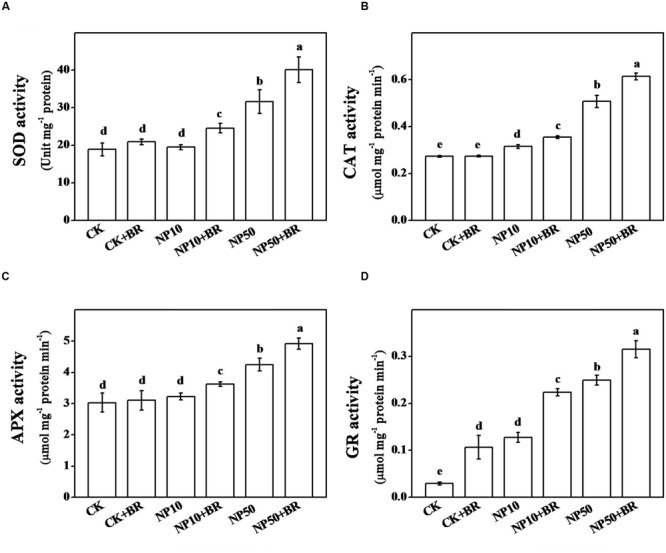
**Response of antioxidant enzymes to individual or combined treatment of brassinosteroid and zinc oxide nanoparticles (ZnO NPs). (A)** Superoxide dismutase (SOD), **(B)** Catalase (CAT), **(C)** Ascorbate peroxidase (APX), and **(D)** Glutathione reductase (GR) activity in tomato roots after 15 days treatment. Tomato seeds after sprouting were placed on ½ MS medium containing 5 nM 24-epibrassinolide (BR) and/or 10 and 50 mg/L ZnO NPs (NP10 and NP50, respectively) and cultured for 15 days before the analysis of activities of antioxidant enzymes. The data shown here are the averages of four replicates, with the standard errors indicated by the vertical bars. The means denoted by the same letter did not significantly differ at a *P* < 0.05, according to Tukey’s test.

Responses of antioxidant enzymes to individual and combined treatment of BR and ZnO NPs have been shown in **Figure 4**. Among four analyzed antioxidant enzymes such as SOD, CAT, APX, and GR, 10 mg/L ZnO NPs increased activities of CAT and GR only, while 50 mg/L ZnO NPs significantly induced activities of all four enzymes. For instance, activities of SOD, CAT, APX, and GR increased by 67.4, 85.8, 40.3, and 747.1% following exposure of seedling to 50 mg/L ZnO NPs for 15 days. Although BR treatment alone increased activity of GR only, combined treatment of BR and ZnO NPs remarkably increased activities of all four enzymes, compared with that of ZnO NPs (low and high) only treatments. These observations further confirm that alleviation of ZnO NPs-induced oxidative stress, is closely associated with enhancement of antioxidant enzyme activity by BR.

In addition, we assessed cellular redox state by analyzing GSH and GSSG contents, and comparing their ratio under different treatments in roots. Compared with the control, high concentration of ZnO NPs increased the contents of GSH, GSSG and GSH+GSSG, but decreased the GSH:GSSG ratio, whereas low concentration of ZnO NPs also increased contents of GSH and GSSG but had no significant effect on the GSH:GSSG ratio. Interestingly, combined treatment with BR and ZnO NPs significantly increased GSH, but decreased GSSG content, resulting in an increased GSH:GSSG ratio compared with that of ZnO NPs only treatments (both low and high). For example, compared with 50 mg/L ZnO NPs only treatment, combined treatment of BR and ZnO NPs increased GSH by 31.9%, but decreased GSSG by 48.5% which eventually increased GSH:GSSG ratio by 156.3%. BR only treatment also significantly increased GSH (32.0%) and decreased GSSG (15.0%), which ultimately increased the GSH:GSSG ratio (55.4%) compared with the control. These results clearly indicate that BR-induced alteration in glutathione helped young tomato seedlings to maintain redox homeostasis under ZnO NPs stress.

### BR Upregulates Transcripts of Various Antioxidant-Related Genes under ZnO NPs Stress

To assess changes in transcripts of antioxidant genes under BR and/or ZnO NPs treatment, we assayed the expression of *Cu/ZnSOD, CAT1, GSH1*, and *GR1* genes in tomato roots. The transcript levels of *Cu/ZnSOD, CAT1, GSH1*, and *GR1* genes were all induced by both low and high ZnO NPs treatments (**Figure 6**). For example, 50 mg/L ZnO NPs treatment upregulated transcripts of *Cu/ZnSOD, CAT1, GSH1*, and *GR1* genes by approximately 1.5, 2.5, 1.8, and 1.9-fold, respectively. Importantly, addition of BR in media further increased the transcript levels of those genes. Thus, highest levels of transcript abundance were observed when seedlings were treated with BR and 50 mg/L ZnO NPs. BR-only treatment also upregulated transcripts of all four genes over control treatment, indicating a positive regulatory role of BR in transcription of those antioxidant-related genes.

**FIGURE 5:**
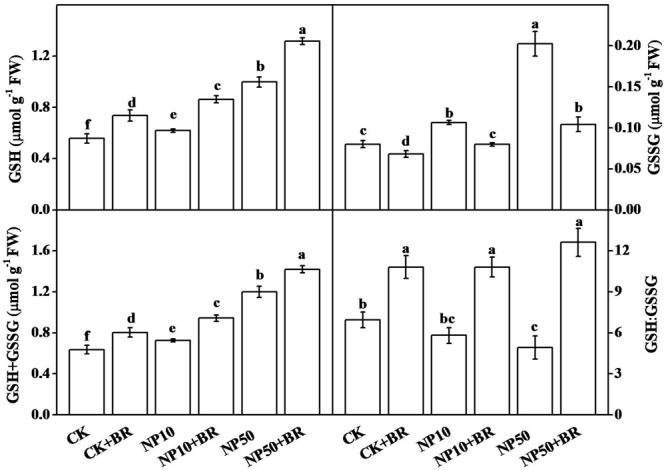
**Effects of zinc oxide nanoparticules (ZnO NPs) and brassinosteroid on glutathione content and redox homeostasis in roots of tomato seedlings.** Tomato seeds after sprouting were placed on ½ MS medium containing 5 nM 24-epibrassinolide (BR) and/or 10 and 50 mg/L ZnO NPs (NP10 and NP50, respectively) and cultured for 15 days before the analysis of glutathione content. The data shown here are the averages of four replicates, with the standard errors indicated by the vertical bars. The means denoted by the same letter did not significantly differ at a *P* < 0.05, according to Tukey’s test. GSH, reduced glutathione; GSSG, oxidized glutathione; GSH+GSSG, total glutathione; GSH:GSSG, ratio between reduced and oxidized glutathione.

**FIGURE 6:**
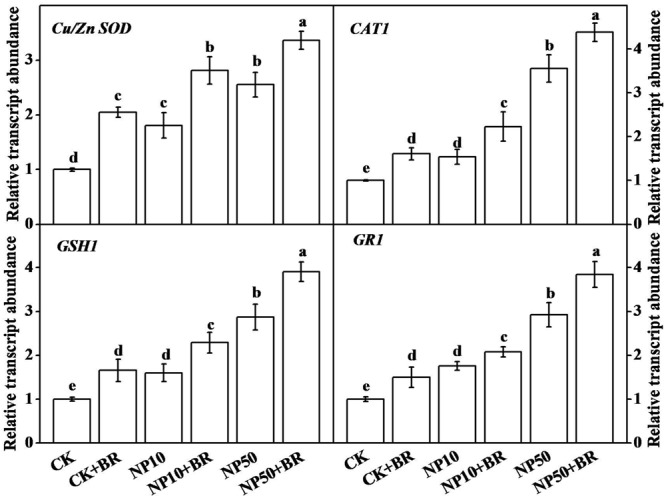
**Expression of antioxidant genes in tomato roots as influence by brassinosteroid and zinc oxide nanoparticles (ZnO NPs).** The expression of genes was analyzed by qRT-PCR after 15 days treatment using gene-specific primer pairs. The data shown here are the averages of four replicates, with the standard errors indicated by the vertical bars. The means denoted by the same letter did not significantly differ at a *P* < 0.05, according to Tukey’s test. BR, 5 nM 24-epibrassinolide; CK, control; NP10, 10 mg/L ZnO NPs; NP50, 50 mg/L ZnO NPs.

### BR Reduces Zn Accumulation under ZnO NPs Stress

**FIGURE 7:**
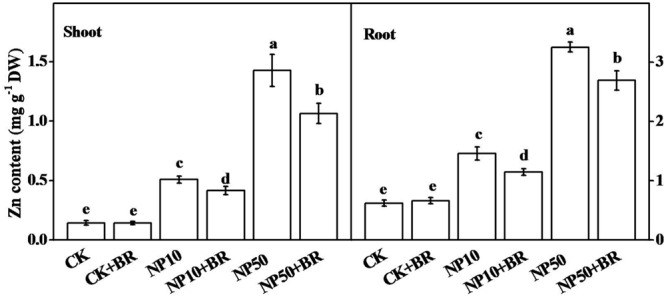
**Interactive effect of zinc oxide nanoparticles (ZnO NPs) and brassinosteroid on accumulation of Zinc in tomato seedlings.** Tomato seeds after sprouting were placed on ½ MS medium containing 5 nM 24-epibrassinolide (BR) and/or 10 and 50 mg/L ZnO NPs (NP10 and NP50, respectively) and cultured for 15 days before the analysis of Zn content. The data shown here are the averages of four replicates, with the standard errors indicated by the vertical bars. The means denoted by the same letter did not significantly differ at a *P* < 0.05, according to Tukey’s test.

To investigate whether BR-induced alleviation of ZnO NPs stress is also associated with changes in Zn accumulation, we quantified Zn content in shoot and root of tomato seedlings following exposure of seedlings to different treatments for 15 days. Zn accumulation in root was much higher than that in shoot under all treatments. In addition, Zn contents in shoot and root of BR only treated seedlings were not different from that of CK seedlings. However, exposure of seedlings to low and high ZnO NPs significantly increased Zn content both in shoot and root (**Figure 7**). The accumulation of Zn was very high in 50 mg/L ZnO NPs treatment, accounting for approximately 9.9 and 5.2-fold higher in shoot and root, respectively, compared with that of CK. Interestingly, Zn accumulation significantly reduced in roots and shoots when BR was applied with ZnO NPs. BR treatment decreased Zn content by 17.9 and 21.4% in shoots and roots, respectively, after exposure to 10 mg/L ZnO NPs, while combined treatment of BR with 50 mg/L ZnO NPs reduced Zn content by 25.4 and 17.3% in shoots and roots, respectively, compared with that in ZnO NPs-only treatment (**Figure 7**). These results indicate that alleviation of ZnO NPs-induced oxidative stress by BR was attributed to BR-mediated reduction in Zn content in shoot and root of tomato seedlings under ZnO NPs stress.

## Discussion

Despite the discrepancy in research findings relating to NPs-induced phytotoxicity, it is now well evident that large scale use of metal-based NPs (MNPs) could appear as a serious threat to crop production and food safety (Chichiriccò and Poma, 2015; Van Aken, 2015). Development of approaches that can alleviate MNPs-induced phytotoxicity may ensure sustainable crop production in the MNPs-polluted marginal lands. In the current study, for the first time, we showed that ZnO NPs-induced phytotoxicity could be alleviated by exogenous application of BR at optimal concentration (5 nM). BR efficiently ameliorated ZnO NPs-induced oxidative stress by promoting antioxidant enzyme activities, redox homeostasis and related gene expression (**Figures 2–6**). In addition, BR decreased Zn content in both shoot and root of young tomato seedling under ZnO NPs stress (**Figure 7**). This study provides promising data that support beneficial role of BR in enhancing plant tolerance to ZnO NPs stress and ensuring food safety.

Phytotoxicity of MNPs is not only due to their small size, large surface area and intrinsic reactivity that allow them to interact with biological macromolecules (protein and nucleic acid), but also associated with the metal constituents. Prior research has shown that MNPs dissolve quickly releasing metal ions that induce production of ROS such as H_2_O_2_ and ^•^OH that are capable to damage vital biomolecules including protein, lipids and DNA (Van Aken, 2015). It is anticipated that ZnO NPs firstly dissolve and then penetrate the cells in the form of Zn^2+^ (Chichiriccò and Poma, 2015). Once they penetrate into the plant, deleterious effects may be produced. In the present study, ZnO NPs exerted a dose-dependent inhibitory effect on tomato growth and biomass accumulation, which was closely associated with accumulation of Zn in plant tissue with increasing concentration of ZnO NPs in culture media (**Figure 1**). ZnO NPs have been shown to reduce root length in corn and cucumber (Zhang et al., 2015), which supports our current observation. It is quite plausible that ZnO NPs inhibit seedling growth concomitantly by directly interacting with biomolecules and also inducing ROS production (Chichiriccò and Poma, 2015). Excessive production of ROS eventually causes an oxidative stress that may lead to cell death (Baxter et al., 2014). To confirm occurrence of oxidative stress upon ZnO NPs treatment, we visualized H_2_O_2_ accumulation and biochemically quantified concentration of H_2_O_2_ and MDA, an important marker of lipid peroxidation (**Figure 3**). Our data clearly indicate that ZnO NPs-induced growth inhibition was associated with excessive levels H_2_O_2_ and MDA. These results are well in accordance with prior reports that showed that MNPs caused plant growth inhibition by inducing oxidative stress in a range of plant species (Dietz and Herth, 2011; Chichiriccò and Poma, 2015).

Besides regulating plant growth and development, phytohormone BR plays a critical role in controlling plant stress responses (Divi et al., 2016). These properties have established BR as a promising plant hormone that could stabilize crop yield under various abiotic stresses such as drought, salinity, extreme temperatures, heavy metals, and organic pollutant stress (Ahammed et al., 2014; Divi et al., 2016). We previously showed that BRs confer Cd tolerance by reducing H_2_O_2_ accumulation and activating antioxidant potential in tomato (Ahammed et al., 2013). Recently, Ramakrishna and Rao (2015) reported that foliar application of BR mitigates ZnO-induced oxidative stress in radish. In conformity with earlier reports, here we observed that addition of 5 nM BR in ½ MS media remarkably increased tomato seedling growth and biomass accumulation under 10 and 50 mg/L ZnO NPs stress (**Figure 2**). This response of BR was highly dose-specific as we noticed an inhibitory effect of BR on seedling growth when applied at 10 nM concentration (data not shown). It is worth mentioning that BRs-regulated plant stress tolerance in a highly dose-dependent manner that greatly differs across the plant species as well as growth stage and tissue type (Ahammed et al., 2014). In tomato, exogenous BR application or endogenous BR level upregulation by overexpression of BR biosynthetic gene *DWARF* results in improved plant growth (Li et al., 2015). BR stimulates cell division, elongation and development that are closely associated with BR-promoted plant growth (Ahammed et al., 2014; Divi et al., 2016).

Reactive oxygen species play a dual role in plant system; on one hand they serve as a critical signaling molecule and on the other hand they cause toxicity when accumulated at high level especially under stress (Baxter et al., 2014). Our prior study showed that H_2_O_2_, a typical ROS, is intricately involved in BR-mediated stress response (Xia et al., 2009). BR triggers transient accumulation of NADPH-based H_2_O_2_ which in turn stimulates antioxidant potential by inducing transcript of antioxidant biosynthetic genes (Cui et al., 2011; Nie et al., 2013; Divi et al., 2016). BR-promoted activities of antioxidant enzymes eventually increase ROS scavenging and thus minimizing oxidative stress under stress (Cui et al., 2011). In the current study, BR treatment significantly reduced accumulation of harmful level of H_2_O_2_ under ZnO NPs stress (**Figure 3A**). In addition to reduction in H_2_O_2_ content, BR-mediated decrease in MDA content revealed that BR efficiently alleviated ZnO NPs-induced oxidative stress in tomato seedlings (**Figure 3C**). This observation was further confirmed by analysis of antioxidant enzyme activities. Supplementation of BR remarkably increased activities of SOD, CAT, APX, and GR under ZnO NPs stress. In plant cells, SOD can rapidly convert $O_{2}^{•–}$ to H_2_O_2_. Once levels of H_2_O_2_ are high due to catalytic activity of SOD or stress induction, some other antioxidative pathways may be activated to diminish excessive H_2_O_2_. CAT is a common antioxidant enzyme that converts H_2_O_2_ to H_2_O and O_2_. Besides, APX can also convert H_2_O_2_ to H_2_O involving ascorbate-glutathione cycle. In addition, GSH is a key antioxidant molecule and can directly decompose H_2_O_2_ by producing oxidant glutathione (GSSG), which is then reduced to GSH by GR (Ma et al., 2015). Our results are in conformity with many earlier reports that showed that application of BR conferred stress tolerance by inducing antioxidant enzyme activities in different plant species (Ramakrishna and Rao, 2012, 2015, p. 29; Ahammed et al., 2013, 2014; Vardhini and Anjum, 2015).

Maintenance of cellular redox homeostasis is critical for normal metabolic processes in plants (Baxter et al., 2014). Under MNPs stress, redox balance is disrupted due to over accumulation of ROS (Chichiriccò and Poma, 2015; Van Aken, 2015). In the current study, GSH:GSSG ratio was significantly decreased under 50 mg/L ZnO NPs stress, indicating an oxidized redox state which is considered harmful for plants. Ag NPs stress also reduced plant biomass but increased accumulation of oxidized glutathione (GSSG, indicative of oxidative stress) in *Triticum aestivum* (Van Aken, 2015). Treatment with BR decreased GSSG content under ZnO NPs stress, which helped young tomato seedlings to maintain a high GSH:GSSG ratio that was almost equivalent to non-stressed seedlings (**Figure 5**). These results are in agreement with earlier reports that showed that BRs could maintain redox homeostasis under Zn and Cd stress in radish and tomato, respectively (Ramakrishna and Rao, 2012; Ahammed et al., 2013).

Brassinosteroid enhances plant tolerance to broad-range stresses, while such enhancement in stress tolerance is correlated with higher expression of genes that are considered as stress markers (Divi et al., 2016). Transcription of antioxidant related genes are generally upregulated under stress (Baxter et al., 2014). To study the molecular bases of the toxicity of ZnO NPs and alleviatory role of BR, we studied expression of antioxidant related genes by using qRT-PCR. In this study, transcripts of *Cu/Zn SOD, GSH1, CAT1*, and *GR1* were induced by ZnO NPs treatment and BR co-application further upregulated their transcript abundance in roots of tomato seedlings. This observation is in line with previous reports, which showed that BR could upregulate expression of various antioxidant related genes in rice and tomato under Cr and Cd stress, respectively (Ahammed et al., 2013; Sharma et al., 2016).

Given that plants are vital components of the food chain, its interaction with NPs essentially have major implications in understanding of environment and public health (Van Aken, 2015). Concerning the connection between BR and food safety, a number of studies have shown that BR could reduce heavy metal accumulation in plants (Ahammed et al., 2013, 2014; Ramakrishna and Rao, 2015; Sharma et al., 2016). In conformity with earlier reports, here we found that BR treatment caused a significant reduction in Zn accumulation under ZnO NPs stress in tomato seedling (**Figure 7**). This implies that BR could also reduce accumulation of metals from MNPs, and thus suggesting a potential implication of BR in ensuring food safety in MNPs-polluted areas. In a recent study, (Chen et al., 2015) showed that NO could alleviate ZnO NPs-induced phytotoxicity by reducing Zn accumulation in rice seedlings. NO is an important signaling molecule that enhances plant tolerance to various environmental stresses. In our earlier study, we found that NO is involved in BR-induced oxidative stress tolerance in cucumber (Cui et al., 2011). Therefore, BR-induced alleviation of ZnO NPs phytotoxicity might also be mediated by regulation of NO (Cui et al., 2011; Chen et al., 2015).

Taken together, this study showed that ZnO NPs induced oxidative stress by increasing accumulation of Zn in plant tissue, which in turn reduced plant growth and biomass accumulation possibly by affecting vital cellular processes. In contrast, treatment with BR alleviated ZnO NPs-induced growth inhibition and oxidative stress by improving antioxidant potential and redox homeostasis. The alleviation of ZnO NPs-induced oxidative stress by BR, was closely associated with upregulation of transcripts of antioxidant genes. BR-induced reduction in Zn content under ZnO NPs stress, provides new prospect for stabilizing crop yield and quality in MNPs-contaminated land. To our knowledge, this study provides the first evidence that BR could alleviate ZnO NPs-induced phytotoxicity in tomato. However, further study involving advanced genetic and molecular tools may help to better understand the detailed molecular mechanisms of BR-induced ZnO NPs tolerance in plants.

## Author Contributions

JZ, ML, GA, and JY planned and designed the research. ML, CL, XB, CH, and HY performed experiments. JZ, ML, and GA analyzed data. JZ, ML, and GA wrote the manuscript.

## Conflict of Interest Statement

The authors declare that the research was conducted in the absence of any commercial or financial relationships that could be construed as a potential conflict of interest.
